# Variability in Donor-Derived Cell-Free DNA Scores to Predict Mortality in Heart Transplant Recipients – A Proof-of-Concept Study

**DOI:** 10.3389/fimmu.2022.825108

**Published:** 2022-02-18

**Authors:** Megan Kamath, Grigoriy Shekhtman, Tristan Grogan, Michelle J. Hickey, Irina Silacheva, Karishma S. Shah, Kishan S. Shah, Adrian Hairapetian, Diego Gonzalez, Giovanny Godoy, Elaine F. Reed, David Elashoff, Galyna Bondar, Mario C. Deng

**Affiliations:** ^1^ Divison of Cardiology, Department of Medicine, Ronald Reagan University of California, Los Angeles (UCLA) Medical Center, Los Angeles, CA, United States; ^2^ Department of Medical Affairs, CareDx Inc., Brisbane, CA, United States; ^3^ Department of Medicine Statistics Core, David Geffen School of Medicine at University of California, Los Angeles (UCLA), Los Angeles, CA, United States; ^4^ University of California, Los Angeles (UCLA) Immunogenetics Center, Department of Pathology and Laboratory Medicine, David Geffen School of Medicine at University of California, Los Angeles (UCLA), Los Angeles, CA, United States; ^5^ Deng Advanced Heart Failure Research Laboratory, Division of Cardiology, Department of Medicine, David Geffen School of Medicine at University of California, Los Angeles (UCLA), Los Angeles, CA, United States

**Keywords:** AlloMap Variability, AlloSure Variability, heart transplantation, donor-derived cell-free DNA (dd-cfDNA), gene expression profiling (GEP), donor specific antibody (DSA), risk prediction, mortality

## Abstract

**Background:**

Over the last decade, expanding use of molecular diagnostics in heart transplantation has allowed implementation of non-invasive surveillance strategies for monitoring allograft health. The commercially available HeartCare platform combines the AlloMap gene expression profiling assay and the AlloSure donor-derived cell-free DNA test (dd-cfDNA). Beyond their established use for assessment of rejection, evidence is building for predictive utility, with the longitudinal AlloMap Variability score previously shown to correlate with the risk of future rejection, graft dysfunction, re-transplantation, or death. In this single-center, retrospective pilot study, we evaluated the performance of a novel AlloSure Variability metric in predicting mortality in a cohort of heart transplant recipients.

**Methods:**

Seventy-two adult heart transplant recipients with at least 3 concurrent AlloMap/AlloSure results were included. Demographic, clinical, imaging, and laboratory parameters were captured. Variability was defined as the standard deviation of longitudinal AlloMap/AlloSure results. A Cox multivariable adjusted proportional hazards model was used to evaluate the variability metrics as predictors of mortality. Associations between AlloMap/AlloSure variability and donor specific antibody (DSA) status were also assessed.

**Results:**

A total of 5 patients (6.9%) died during a median follow-up of 480 days. In a univariate Cox proportional hazards model, higher AlloSure variability (HR 1.66, 95%CI 1.14 – 2.41), but not AlloMap variability or the cross-sectional AlloSure/AlloMap results was associated with increased mortality risk. Longitudinal AlloSure variability was also higher among patients with both preformed DSA and those developing *de novo* DSA.

**Conclusion:**

Our results suggest that increased variability of dd-cfDNA in heart transplant patients is associated with both mortality risk and the presence of donor specific antibodies. These findings highlight the added value of longitudinal data in the interpretation of AlloMap/AlloSure scores in this population and open the door to larger studies investigating the utility of these metrics in shaping post-transplant clinical care paradigms.

## Introduction

In parallel to the clinical maturation of heart transplantation over the last 50 years, rejection testing has been revolutionized within the systems biology paradigm triggered by the Human Genome Project. The development of the first FDA-cleared diagnostic and prognostic leukocyte gene expression profiling (GEP) biomarker test in transplantation medicine (AlloMap) and its inclusion in international evidence-based medicine guidelines (1, 2) prompted molecular re-classification of intragraft biology [myocyte injury, acute cellular rejection (ACR), antibody-mediated rejection (AMR)] and stimulated research into other technologies for non-invasive detection of cardiac allograft injury. These efforts produced the first donor organ-specific cardiac injury marker based on donor-derived cell-free DNA (dd-cfDNA), further enhancing the clinical utility of non-invasive monitoring by combining two complementary non-invasive blood-based measures, host immune activity-related risk of acute rejection as well as cardiac allograft injury (3, 4).

During the early years of clinical implementation of noninvasive monitoring with GEP, we observed an association of low variability of longitudinal scores and the clinical stability of the individual transplant recipient. We hypothesized that the variability of GEP scores within individuals may predict risk of future allograft events and tested this hypothesis by analyzing the Invasive Monitoring Attenuation through Gene Expression (IMAGE) study dataset of 602 heart transplant recipients (5). In the multivariate analyses, AlloMap score variability, but not ordinal scores or scores over threshold, was independently associated with future clinical events such as rejection, graft dysfunction, re-transplantation or death. These findings have subsequently been validated in an independent European cohort by Crespo-Leiro and colleagues (6). For the management of heart transplant patients, these results suggest that a recipient predicted to be at low risk for future events may become a candidate for minimization of immunosuppression. Conversely, an individual predicted to be at higher risk for future events may receive further evaluation to detect possible underlying causes of the variability such as overlooked infections or noncompliance to medications (7).

Leveraging the fact that an organ transplant is also a genome transplant, the development of methods to reliably quantify dd-cfDNA have added another dimension to non-invasive post-transplant surveillance. The clinical validity dd-cfDNA as non-invasive marker of allograft injury has been demonstrated in studies across the spectrum of solid organ transplantation, including kidney, lung, and heart transplant recipients (8–13). Beyond its role as a marker of allograft injury, there is emerging evidence that dd-cfDNA may play a mechanistic role in the activation of inflammatory pathways, predict the development of *de novo* DSA, and identify patients at risk of adverse long-term clinical outcomes (14–16). The integration of dd-cfDNA-based graft injury assessment with AlloMap surveillance of immune system “quiescence” thus represents an informative, multimodal, non-invasive strategy for longitudinal surveillance of heart transplant recipients, an approach we have utilized at our center since 2018 with the HeartCare platform (AlloMap GEP and AlloSure dd-cfDNA; CareDx Inc., Brisbane, CA).

Building on this single-center experience and our previous development and validation of the AMV score, we postulated that added predictive power could be derived *via* assessment of AlloSure Variability (ASV). Given the clinical phenotype we observed in patients with increased AMV in our previous studies, we hypothesize that fluctuating molecular injury patterns, as assessed by longitudinal variability in AlloSure results, may enhance the identification of patients at risk for adverse long-term clinical outcomes.

## Materials and Methods

### Study Design

We conducted a single-center, retrospective cohort study, utilizing clinical and laboratory data from heart transplant recipients followed at the University of California, Los Angeles (UCLA). The source of the clinical and laboratory data used in this analysis was the electronic medical record (EMR) at UCLA. Relevant data collected included demographic information, pre-transplant data (including organ donor characteristics and relevant pre-transplant medical history), biopsy results (including indication and histologic diagnosis), post-transplant outcomes (including primary graft dysfunction, preformed or *de novo* (dn) donor specific antibody (DSA), development of cardiac allograft vasculopathy (CAV), clinical/histologic rejection, allograft failure, re-transplantation, and death), and all relevant laboratory/imaging data (including AlloSure/AlloMap results).

### Study Participants

Seventy-two (72) adult heart transplant recipients followed at UCLA and undergoing non-invasive surveillance with AlloMap/AlloSure were included. Our current protocol allows transition to a strategy of non-invasive surveillance at 2 months post-transplant for those patients not meeting high-risk criteria (allograft dysfunction, prior AMR or recent ACR, highly sensitized patients, or those felt to be at substantially increased risk of rejection). Patients with contraindications to surveillance biopsies, including those with coagulopathies necessitating chronic anticoagulation are also routinely monitored with non-invasive surveillance and are included in our cohort. All subjects were adult heart transplant (HTx) patients who underwent combined AlloMap/AlloSure testing between January 1, 2018 and December 31, 2020. The follow-up period ended on April 30, 2021. Patients were included if they were: 1) recipients of first or repeat heart transplant receiving clinical follow-up at the UCLA Heart Transplant Clinic, 2) 18 years of age or older, and 3) had at least 3 paired AlloSure/AlloMap results available. The date of the first paired AlloSure/AlloMap draws was considered the study enrollment date. Multiorgan transplant recipients (heart/kidney, heart/liver, heart/lung, etc.) were excluded. This retrospective analysis was approved by the UCLA IRB (Protocol 18-002046). Given the retrospective nature of the study, lack of interaction with or risk to participants, and de-identified nature of data assessment, the UCLA IRB granted a waiver of consent for this study. The Investigators ensured that this study was conducted in accordance with the principles of the Declaration of Helsinki.

### HLA Typing and Evaluation of HLA Antibodies

HLA typing of patients and donors was performed for HLA-A, -B, -C, -DRB1, -DQB1, -DQA1, -DPA1, and -DPB1 loci using LABType SSO (One Lambda, Canoga Park, CA). For antibody assessment, undiluted sera samples were treated with DTT and tested for HLA antibodies using the IgG-SAB Assay from One Lambda (Canoga Park, CA) as previously described (17). Antibodies were considered positive if the MFI >1000 for HLA-A, B, DR, DQ and >2000 was used for HLA-C and DP.

### Outcome Measures

In this proof-of-concept pilot analysis of the predictive utility of ASV, mortality was utilized as the primary endpoint. Our rationale for the selection of this endpoint included its clinical relevance and an event rate in our cohort that would allow for the proposed analysis. Additionally, we investigated how variability scores differed in patients stratified by DSA status, including those without DSA, those with preformed DSA, and those developing *de novo* DSA (dnDSA) during the study period.

### Statistical Methods and Analysis

Demographic and clinical characteristics were summarized using descriptive measures. AlloMap and AlloSure variability estimates were computed using either the standard deviation of the 3 most recent paired results (3-value variability) or all available results (all-value variability), depending on the analysis performed. For summary measures of AlloSure ordinal scores, we specified when results were below the limit of detection; for the purposes of calculating standard deviations to quantify variability, all AlloSure scores below the limit of detection were treated as 0.10%. Univariate Cox proportional hazards models for mortality were constructed for each variable. AMV/ASV were mean centered, and for analysis purposes, ASV was rescaled (multiplied by 10, ASV*10) since a 1 unit increase in ASV spanned nearly the entire range of values. The time to mortality outcome measure was computed as the difference between date of mortality (or study censor date) and time of first pair of AM/AS draw. The time between original transplant and time to first draw was recorded as “Time Post-Transplant” and included in our summary table. The performance of this model, which included the covariates significant on univariate analysis (p<0.1) and the AlloSure variability metric, was evaluated using Harrell’s concordance index. The possibility of identifying a specific cut-point or threshold which best identified mortality outcomes was also explored using the ‘rpart’ package in R, which uses recursive partitioning to create several subpopulations in order to maximize fit criteria specifically for survival data (18). Since the initial trees are known to be overfit and unlikely to validate, we also used the suggested ‘prune’ function which aids in combating overfitting the data by using cross-validation. AMV/ASV values were compared with DSA [three groups: no DSA (n=48), dnDSA (n=15), and preformed DSA (n=9)] using the Kruskal-Wallis test. AMV/ASV was also compared between patients with dnDSA diagnosis to those without using the Wilcoxon test. Statistical analyses were run using R V 3.6.1 (www.r-project.org, Vienna, AU) and p-values <0.05 were considered statistically significant.

## Results

### Patient Cohort Characteristics

A total of 72 patients were included in the study cohort. Average age at the time of transplant was 49.1 years (SD 14.3) and enrollment (initial AM/AS test) occurred at a median of 112.5 days (IQR: 73.5 – 277) after transplant. In our study cohort, 62.5% of patients were male, 45.8% were white, and 72.2% had non-ischemic cardiomyopathy as the etiology of heart failure. Most patients (82%) were enrolled within the first year after heart transplant. A total of 7 patients (9.7%) were repeat transplant recipients. Induction therapy was used in 43.1%. Mean baseline left ventricular ejection fraction at the time of enrollment was 64.1% (SD 7.8). Median follow-up time after first AM/AS result was 480 days (IQR: 244 - 859). Sixteen patients had biopsy-proven rejection events (ACR ≥ 2R and/or pAMR > 0) after transplantation; only one patient had biopsy-proven rejection during the study period, and the rest of the rejections occurred prior to study enrollment and initiation of AM/AS surveillance. Additional demographics and clinical characteristics are detailed in **(** **Table 1** **)**.

**Table 1:** Characteristics of the study population.

Patient Characteristics	Mean (SD) or Frequency (%) of (n=72)
**Age (years)**	49.1 (14.3)
**Male Gender**	45 (62.5%)
**Race^*ψ*^**	
Asian	7 (9.7%)
Black	10 (13.9%)
Other	22 (30.6%)
White	33 (45.8%)
**Hispanic Ethnicity^*ψ*^**	26 (36.1%)
**Transplant Indication**	
CAD	15 (20.8%)
Nonischemic cardiomyopathy	50 (69.4%)
Re-transplantation	7 (9.7%)
**Use of Ventricular Device Pre-Transplant**	15 (20.8%)
**Induction Therapy**	
ATG	8 (11.1%)
Basiliximab	23 (31.9%)
None	41 (56.9%)
**Hypertension**	40 (55.6%)
**Diabetes**	33 (45.8%)
**Dyslipidemia**	51 (70.8%)
**Renal Insufficiency*∫***	21 (29.2%)
**Cancer**	3 (4.2%)
**Baseline LVEF (%)**	64.1 (7.8)
**Time Post-Transplant (days)**	270.7 (420.2)
**CMV Status*^§^***	
D not available, R+	1 (1.4%)
Not available	4 (5.6%)
D+R-	11 (15.3%)
D+R+	33 (45.8%)
D-R-	8 (11.1%)
D-R+	15 (20.8%)
**Immunosuppression Regimen**	
Tacrolimus/MPA/Prednisone	72 (100%)
**DSA Status**	
No DSA	48 (66.7%)
Preformed DSA	9 (15.3%)
dnDSA	13 (20.8%)
Preformed and dnDSA	2 (2.7%)
**Biopsy-Proven Rejection (patients)**	
ACR ≥2R	4
pAMR > 0	11
Both ACR ≥2R and pAMR > 0	1
No Rejection	56

**^y^** Race or ethnic group was self-reported.

^**∫**^Mean ( ± SD) serum Creatinine for patients categorized as having renal insufficiency was 2.03 ± 1.01 mg/dL.

**^§^** D, Donor; R, Recipient; (+)/(-) reflect presence/absence of serum IgG Ab.

### AlloMap/AlloSure Results

**Figure 1:**
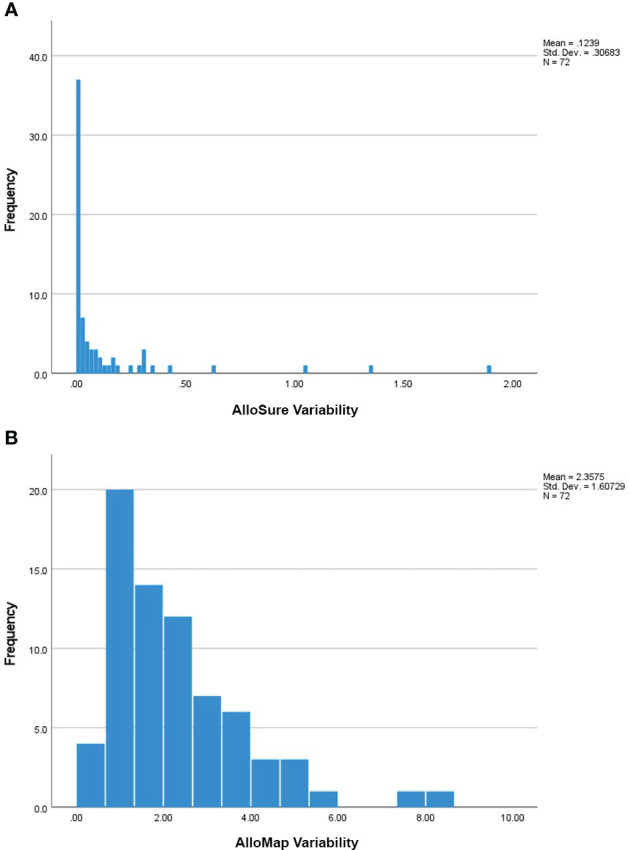
**(A)** Frequency of the Calculated AlloSure Variability Measures: Frequency distribution of AlloSure variability measures for all enrolled patients (n=72). AlloSure score variability was defined as the standard deviation of at least 3 sequential AlloSure results obtained post-transplant. 35 patients had no calculated variability (ASV = 0) due to longitudinal scores being persistently below the limit of detection. The remainder (n=37) had a median AlloSure variability of 0.08 (IQR: 0.04 – 0.29). For subsequent analyses, ASV is reported as ASV * 10 to allow easier interpretation of the results. **(B)** Frequency of the Calculated AlloMap Variability Measures: Frequency distribution of AlloMap variability measures for all enrolled patients (n=72). AlloMap score variability in this study was defined as the standard deviation of at least 3 sequential AlloMap results collected after transplantation. Mean AlloMap variability in our cohort was 2.36 (SD 1.61).

Patients in this cohort had a median of 6 (IQR: 4-9) paired AlloSure/AlloMap results available for analysis. Median time between consecutive results was 40.8 days (IQR: 31.5-66.1). Thirty-five (35) patients had longitudinal AlloSure results below the limit of detection while the remainder demonstrated a median AlloSure score of 0.17% (IQR: 0.12%-0.45%) and a median all-value variability (ASV) of 0.08 (IQR: 0.04 – 0.29) **(** **Figure 1A** **)**. The average AlloMap score in this cohort was 33.4 (SD: 2.8) with an average all-value variability (AMV) of 2.36 (SD: 1.61) **(** **Figure 1B** **)**.

### Association of AlloMap/AlloSure Variability Scores With Mortality

**Figure 2:**
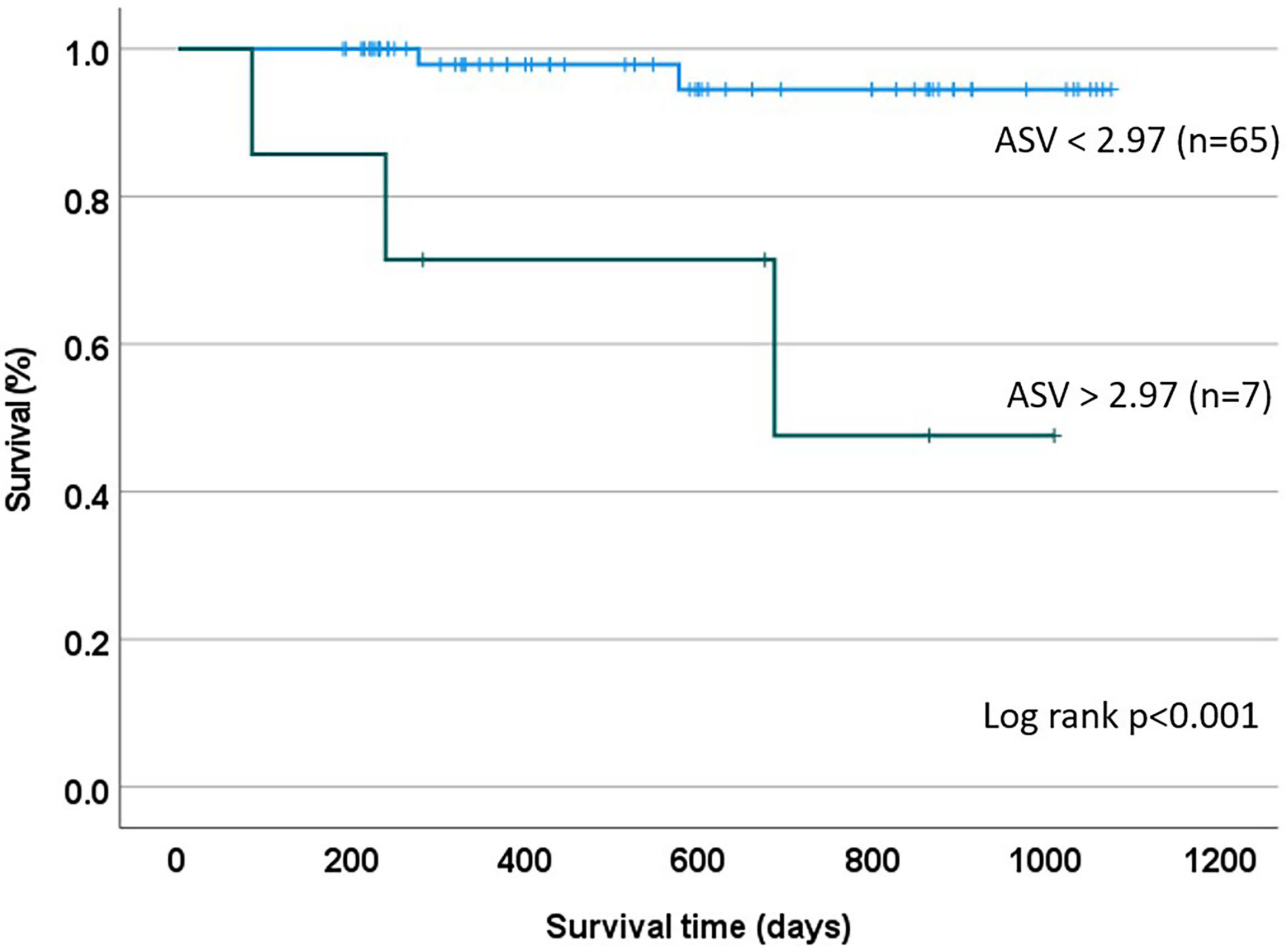
Kaplan Meier Estimate based on CART Analysis: A recursive partitioning and cross-validation algorithm was utilized to identify an ASV threshold of 2.97 as being best suited for discrimination between patients at high and low mortality risk.

A total of 5 patients (6.9%) died during the follow-up period, occurring a median of 277 days (IQR: 162-632) after the first paired AM/AS result. Two of the five were re-transplant patients and one had a history of prior biopsy-proven rejection. Patients who died had higher mean 3-value AMV (1.83, SD:1.98) and median ASV*10 (3.24, IQR: 0.00 – 3.62) compared to survivors, who had an AMV of 1.62 (SD: 1.42) and an ASV*10 of 0.00 (IQR: 0.00 – 0.46), though these differences were not statistically significant. There was no statistically significant difference in AMV or ASV in patients with or without a history of biopsy-proven rejection. Cohort survival was estimated (KM method) at 95% at a median follow-up time of 480 days. In a univariate Cox proportional hazards model, only the 3-value ASV*10 metric, but not 3-value AMV, dnDSA, or ordinal AM/AS scores were associated with time to death. In the final time-to-event multivariable model (including those covariates with p < 0.1 in the univariate model), no individual parameter demonstrated an independent association with mortality, though the ASV metric approached significance (HR 1.51, 95%CI: 0.96 – 2.38, p = 0.074) **(** **Table 2** **)**. In a survival classification and regression tree (CART) model, an ASV*10 cut point of 2.97 was identified as splitting the patients into high and low risk groups with regards to mortality risk (p<0.001 using the log-rank test) **(** **Figure 2** **)**.

**Table 2:** Univariate and multivariate Cox proportional hazards model for mortality.

Clinical Characteristics	Univariate HR (95% CI)	p-value	Multivariate HR (95% CI)	p-value
Age	0.99 (0.94-1.05)	0.84		
Male gender	1.10 (0.18-6.61)	0.916		
Black Race	4.53 (0.75-27.43)	0.1		
Indication: retransplant (y/n)	5.08 (0.85-30.46)	0.075	2.61 (0.36 – 19.13)	0.346
Induction (y/n)	0.99 (0.16-6.00)	0.995		
HTN	0.59 (0.10-3.52)	0.56		
Diabetes	0.67 (0.11-4.03)	0.661		
Dyslipidemia	1.09 (0.12-9.91)	0.937		
Renal insufficiency	1.29 (0.21-7.81)	0.782		
Baseline LVEF	1.07 (0.91-1.25)	0.429		
History of rejection	0.73 (0.08-6.59)	0.782		
Time post-transplant	1.00 (0.99-1.01)	0.664		
**Molecular Parameters**	**Univariate HR (95% CI)**	**p-value**	**Multivariate HR (95% CI)**	**p-value**
AM Variability	1.16 (0.68-1.99)	0.588		
AS Variability*10	1.66 (1.14-2.41)	0.009	1.51 (0.96 – 2.38)	0.074
dnDSA (n=15)	4.98 (0.83-29.84)	0.079	4.44 (0.68 – 29.04)	0.120
Peak AM	1.01 (0.67-1.51)	0.977		
Peak AS	1.07 (0.54-2.12)	0.837		
Last AM	1.12 (0.80-1.57)	0.519		
Last AS	1.08 (0.24-4.82)	0.923		
			Concordance (Harrell’s)	0.781

In a univariate Cox proportional hazards model, only the ASV metric was associated with mortality. In the final time-to-event multivariate model, no covariates retained their statistically significant association with the outcome. The following terms are abbreviated as: Hypertension (HTN), Left Ventricular Ejection Fraction (LVEF), de novo DSA (dnDSA), AlloMap (AM), and AlloSure (AS).

### Association of AlloMap/AlloSure Variability Scores With DSA Status

Of the 72 patients included in the study, 48 (66.7%) did not have DSA at any time point; 9 had preformed DSA (12.5%), 13 (18%) had dnDSA, and 2 (2.7%) had both preformed DSA and dnDSA that was later developed to additional HLA specificities **(** **Table 1** **)**. For downstream analyses, these two were grouped with the dnDSA patients. Among the sixteen patients with a history of biopsy-proven rejection, three patients had preformed DSA and four patients had dnDSA. The median time from transplant to dnDSA detection was 329 (39-827) days. There were significant differences in all-value ASV*10 among patients within these DSA groups, with those patients developing dnDSA having the highest median ASV*10 (1.70, IQR: 0.30 – 3.10) compared to those without DSA (0.00, IQR: 0.00 – 0.45) or existing DSA (0.20, IQR: 0.00 – 0.70), p = 0.001 **(** **Table 3** **)**. Differences were also seen in all-value AMV between these groups, with the highest mean AMV seen in patients without DSA (2.68 ± 1.66) compared to either those with dnDSA (2.22 ± 1.40) or those with existing DSA (0.86 ± 0.31), p < 0.001.

**Table 3:** All-value AlloSure/AlloMap variability and DSA status.

	No DSA (n=48)	dnDSA (n=15)	Preformed (n=9)	p-value
Mean AMV (SD)	2.68 (1.66)	2.22 (1.40)	0.86 (0.31)	<0.001
Median ASV*10 (IQR)	0.00 (0.00 – 0.45)	1.70 (0.30 – 3.10)	0.20 (0.00 – 0.70)	0.001

The opposite pattern was seen for all-value AMV. Patients with dnDSA had higher all-value ASV than either patients with preformed or absent DSA.

To determine if the presence of dnDSA was temporally correlated with AMV/ASV, 3-value variability in patients who developed dnDSA was compared with the all-value variability among patients without DSA. The all-value variability metric was used for this comparison in patients without DSA because of the absence of an event (detection of dnDSA). AMV in patients before dnDSA detection (n = 6) was lower (1.34 *vs* 2.68, p = 0.024) while ASV*10 trended higher, but the difference was not statistically significant (0.70 *vs* 0.00, p = 0.380). Median time between the last paired AM/AS result and dnDSA detection in this group was 162 days (81 – 305). Conversely, when comparing AMV/ASV in patients after dnDSA detection (n = 10) to patients without DSA, AMV did not significantly differ (2.13 *vs* 2.68, p = 0.123) while ASV*10 was significantly higher (0.48 *vs* 0.00, p = 0.014) **(** **Table 4** **)**. Among these patients, median time between dnDSA detection and the first subsequent AM/AS result was 84 days (IQR: 32 – 225).

**Table 4:** Relationship between AMV/ASV and dnDSA detection.

Measure	No DSA (all-value, n=48)	Before dnDSA (3-val, n=6)	p-value
Mean AMV (SD)	2.68 (1.66)	1.34 (0.87)	0.024
Median ASV*10 (IQR)	0.00 (0.00 – 0.45)	0.70 (0.00 – 2.73)	0.380
**Measure**	**No DSA (all-value, n=48)**	**After dnDSA (3-val, n=10)**	**p-value**
Mean AMV (SD)	2.68 (1.66)	2.13 (1.67)	0.123
Median ASV*10 (IQR)	0.00 (0.00 – 0.45)	0.48 (0.12 – 2.04)	0.014

Only those patients with at least 3 AM/AS measurements prior to or after dnDSA detection included. Comparison using Wilcoxon rank sum test. Patients had lower AMV prior to dnDSA detection and higher ASV after dnDSA detection.

## Discussion

In the current proof-of-concept pilot study, we postulated that a novel ASV metric would have predictive power in patients following heart transplantation. Specifically, we hypothesized that increased ASV identifies a population of patients who are experiencing cycles of molecular injury that may predispose them to an enhanced risk of adverse clinical events. Our initial findings support this hypothesis, demonstrating that ASV identifies patients at an increased risk of mortality **(** **Table 2** **)**. Interestingly, ASV was a more potent predictor of mortality than the ordinal AlloMap/AlloSure scores, rejection history, DSA status, or other traditional clinical risk factors, suggesting that longitudinal assessment of AlloSure scores may have a novel and complementary role in the care of heart transplant recipients **(** **Table 2** **)**. Although this association was not significant in the multivariate model, the finding merits re-evaluation in a larger cohort, where a higher event rate may allow more definitive assessment. Using a recursive partitioning algorithm, we also explored the feasibility of defining a threshold ASV (equal to 2.97) that could stratify patients into high- and low-risk subpopulations, an approach that may allow individualizing post-transplant surveillance strategies if these findings are confirmed in larger studies **(** **Figure 2** **)**.

We also observed associations between AlloSure Variability and DSA status, similar to previously reported data in both heart and kidney transplant recipients (19, 20). The finding that all-value ASV is higher in patients who develop dnDSA compared to those who remain DSA-negative supports the idea that pathogenic and consequential processes are associated with allorecognition (21). The increase in ASV seen in patients after dnDSA detection also fits this paradigm, with variability potentially imparted by either augmentation of immunosuppression or rejection therapy **(** **Table 4** **)**. The significance of the intermediate ASV seen in patients with pre-formed DSA is uncertain but may reflect the spectrum of pathogenicity seen in patients with pre-formed antibodies **(** **Table 3** **)**. Conversely, the highest AMV was seen in those patients without DSA, a somewhat unexpected observation **(** **Table 3** **)**. Given that the gene set utilized by the AlloMap assay was selected specifically to identify GEP patterns associated with ACR, the significance of lower AMV in patients with pre-formed DSA and those who develop dnDSA merits further investigation **(** **Tables 3**, **4** **)**.

Limitations of this study include its single-center, retrospective design, as well as its relatively small sample size and low event rate. Time to mortality was evaluated as the primary endpoint in this proof-of-concept analysis. While this is the largest study of ASV to our knowledge, the small number of mortality events may have led to overfitting of the regression model. Furthermore, our study population contained a relatively high proportion of re-transplant recipients (9.7%), a known risk factor for reduced post-transplant survival (22); however, despite having two retransplant recipients among the five patients who died, re-transplantation was not an independent risk factor for mortality in our multivariate model. To accommodate our study inclusion criteria, we used a 3-value AMV calculation, a modification that precludes comparison of its performance to previously published literature (6, 7). Studies utilizing larger datasets are needed to both validate our findings and investigate whether some combination of ordinal scores and variability metrics will further enhance the predictive performance of ASV.

Once validated in larger studies, the ASV metric can further enhance and complement the information derived from the use of these assays in longitudinal surveillance of heart transplant recipients. Interpretation of ordinal scores and trajectories can provide actionable data on the likelihood of active injury or rejection, while variability measures could risk-stratify patients, identify candidates for immunomodulation, and help determine frequency of clinical, laboratory, and echocardiographic follow-up.

## Conclusion

In this proof-of-concept study utilizing a single-center, retrospective cohort of heart transplant recipients, we have shown that variability in serial AlloSure values over time may help identify patients at increased risk of mortality. We also observed increased variability in those patients with dnDSA. Our findings further expand on the potential clinical utility of surveillance AlloSure (dd-cfDNA) testing in heart transplant recipients, however, additional large-scale studies are needed to validate these results.

## Data Availability Statement

The raw data supporting the conclusions of this article will be made available by the authors, without undue reservation.

## Ethics Statement

The studies involving human participants were reviewed and approved by Medical IRB 1 UCLA - Chair Dr. Clemens. Written informed consent for participation was not required for this study in accordance with the national legislation and the institutional requirements.

## Author Contributions

MD designed the original hypothesis and study, participated in data retrieval, analysis, and interpretation, and participated in manuscript writing. MK, GS, and GB co-developed the original hypothesis and study, participated in data retrieval, analysis, and interpretation, and participated in manuscript writing. TG and DE were co-responsible for statistical analysis, and interpretation, and participated in manuscript writing. MH and ER were co-responsible for immunogenetics data analysis and interpretation and participated in manuscript writing. IS, KaS, KiS, AH, DG, and GG participated in data retrieval, analysis, and interpretation, and participated in manuscript writing. Authorship was determined in accordance with the ICMJE guidelines and other contributors were acknowledged. All authors contributed to the article and approved the submitted version.

## Funding

This study received funding from CareDx Inc. Additional funding includes UCLA NIH R211R21HL120040-01 (MCD) (PI Deng), UCLA R01 (PI Weiss, Joint PI Deng), UCLA R01 (PI Ping, Co-I Deng), University of Pennsylvania R01 1R01AI144522-01A1 (Contact PI Keating, Co-PI Deng), UCLA DOM Internal Funds and the Advanced HF Research Gift to Columbia University (Philip Geier, John Tocco and Robert Milo), Advanced HF Research Gift to UCLA (Larry Layne, Juan Mulder, James and Candace Moose, and Peter Schultz), UCLA RO1AI135201 and R21AI156592 (Reed),U19AI128913 (PI: Reed), 1R01AI135201 (PI: Reed), and 1R01 (PI: Deng (Contact-PI)). The funder was not involved in the study design, collection, analysis, interpretation of data, the writing of this article or the decision to submit it for publication.

## Conflict of Interest

GS is employed by CareDx Inc. MD serves on the national scientific advisory board of CareDx Inc.

The remaining authors declare that the research was conducted in the absence of any commercial or financial relationships that could be construed as a potential conflict of interest.

## Publisher’s Note

All claims expressed in this article are solely those of the authors and do not necessarily represent those of their affiliated organizations, or those of the publisher, the editors and the reviewers. Any product that may be evaluated in this article, or claim that may be made by its manufacturer, is not guaranteed or endorsed by the publisher.
